# Association of Surfactant Protein D Single Nucleotide Polymorphisms rs721917, rs2243639, rs3088308 with Recurrent Aphthous Stomatitis in Pakistani Population

**DOI:** 10.3390/genes14051119

**Published:** 2023-05-22

**Authors:** Zainab Rizvi, Nakhshab Choudhry, Aamir Jamal Gondal, Nighat Yasmin

**Affiliations:** 1 Department of Oral Pathology, de’ Montmorency College of Dentistry, Lahore 54000, Pakistan; zainabrizvi514@gmail.com; 2 Department of Biochemistry, King Edward Medical University, Lahore 54000, Pakistan; drnbchoudhry@gmail.com; 3 Department of Biomedical Sciences, King Edward Medical University, Lahore 54000, Pakistan; ajgondal119@gmail.com

**Keywords:** recurrent aphthous stomatitis, surfactant protein D, SNP, Pakistan

## Abstract

Recurrent aphthous stomatitis (RAS) is a benign ulcerative condition, defined by the recurrent formation of non-contagious mucosal ulcers. Surfactant protein D (SP-D) is secreted frequently at surfaces exposed directly to body fluids. This study aims to investigate the association of SP-D single nucleotide polymorphisms (SNPs) with the onset of RAS. Blood samples from 212 subjects (106 cases/controls each) were collected during 2019 and genotyped for SP-D SNPs (rs721917, rs2243639, rs3088308) by polymerase chain reaction and restriction fragment length polymorphism followed by 12% polyacrylamide gel electrophoresis. Minor aphthous (75.5%) was the commonly observed ulcer type as compared to herpetiform (21.7%) and major aphthous ulcers (2.8%). A family history of RAS was reported in 70% of cases. RAS was found significantly associated with rs3088308 genotypes T/A (95% (Cl): 1.57–5.03, *p* = 0.0005), A/A (95% (Cl): 1.8–6.7, *p* = 0.0002), T-allele (95% (Cl): 1.09–2.36, *p* = 0.01), A-allele (95% (Cl): 1.42–3.91, *p* = 0.01), rs721917 genotype T/T (95% (Cl): 1.15–25.35, *p* = 0.03), and T-allele (95% (Cl): 1.28–3.10, *p* = 0.002). Female gender and obese body mass index (BMI) were significantly associated with rs3088308 genotypes T/A (95% (CI): 1.89–15.7, *p* = 0.001), T/T (95% (Cl): 1.52–11.9, *p* = 0.005), A-allele (95% (Cl): 1.65–7.58, *p* < 0.001), and T-allele (95% (Cl): 1.4–10.1, *p* <0.001) and rs721917 genotype T/T (95% (CI) = 1.3–33, *p* = 0.02), respectively. This study describes the association of SP-D SNPs (rs721917, rs3088308) with RAS in the Pakistani population.

## 1. Introduction

The oral cavity is a unique anatomical site consisting of different parts that work together to safeguard the oral mucosa from the harmful environment. Oral mucosal lesions constitute a broad spectrum of clinical manifestations in the form of different signs and symptoms that can be challenging for general physicians and dentists to diagnose accurately. These lesions can be a source of pain and uneasiness for patients, thereby preventing them from carrying out routine oral activities, such as mastication, swallowing, fluid intake, and speech [1,2,3,4]. An oral ulcerative lesion is defined as a breach in the epithelium and defect in connective tissue lying underneath the mucosa or epithelium [5]. Three main classes of ulcerative lesions of the oral cavity have been identified; acute, chronic, and recurrent ulcers [1,5].

Recurrent aphthous stomatitis (RAS) is a painful localized mucosal ulcerative condition, characterized by recurring episodes of tender, isolated, or abundant ulcerations limited to the mucosa of the oral cavity [6,7]. RAS is categorized into different groups on the basis of the spectrum of disease [8]. The characteristic presentation of RAS includes the recurrence of solitary or numerous, small or large, ovoid or irregular painful ulcerations in the oral cavity, encircled by an erythematous margin and enveloped by a greyish white or yellowish pseudo-membrane [8,9,10]. There are three clinical variants of aphthous ulcer based on the onset, incidence, duration, number, size, and healing fate of the ulcer: minor aphthae, major aphthae, and herpetiform aphthae [11,12,13,14]. These aphthous ulcers follow a clinical sequence of four stages (prodromal, pre-ulcerative, ulcerative, and healing) that have proved to be very facilitating in diagnosis and management, keeping in mind the fact that RAS is a disorder of unidentified pathogenesis [7,15,16]. Epidemiological studies reported a high prevalence of RAS affecting 2–66% of the global population with a lifetime predominance of 38.7% in men and 49.7% in women [17,18]. Global data show geographic variations in the frequency of RAS incidence, such as 64% in the geriatric Indian population, 27.17% in China, 78% in Jordan, 44.5% in Iran, 24.9% in Brazil, 40.81% in the U.S., 28.2% in Iraq, and 22.5% in Pakistan [19,20,21,22,23,24,25,26].

Among the several proposed risk factors for RAS, a strong association of genetic predisposition has been suggested as 30–40% of cases have shown a positive family history [9,14,18]. Polygenic mode of inheritance or autosomal recessive inheritance pattern are considered to be involved in the RAS development with a significant contribution from environmental factors [14]. There is a 90% probability of RAS development in children of parents who have been diagnosed with RAS once or more than once in their lifetime [13]. If only one of the parents has RAS, the chance of developing the disease reduces to 20%. The individuals with the familial predisposition of RAS show onset of RAS in the early years of their life [12,27]. Moreover, genetically mediated immune system disturbances are implicated in the RAS development [27]. A study conducted for genetic screening of monogenetic inheritance demonstrated the mutations of STAT1, PTPN22, TNFAIP3, and DNASE1L3 in the RAS infant population [28]. The genetic polymorphisms in human leukocyte antigens have also been linked to RAS. Geographical locations and racial factors do have an impact on these polymorphisms [12,18,27]. Several reports have described the association of single nucleotide polymorphisms (SNPs) of different factors with RAS, such as E-selectin, Toll-like receptor 4 (TLR-4), interleukin-10 (IL-10), IL-1β and tumor necrosis factor alpha (TNFα), and IL-6 and matrix metalloproteinase 9 (MMP-9) [18,29,30,31,32].

Surfactant proteins (SPs) are categorized into a family of molecules (collectins) related to the immune system that were initially identified in the pulmonary region [33]. Among different SP variants, SP-D and SP-A are known to be involved in the activation of macrophages leading to the regulation of inflammation [34]. The presence of SP-D and SP-A have been demonstrated outside the pulmonary system, such as in lacrimal and salivary glands, pancreas, nervous system, renal system, cardiovascular system, and reproductive tract [35,36]. The expression of SP-D and SP-A in the duct epithelial cells of salivary glands represents a defense mechanism of oral mucosa that is constantly exposed to infectious agents [34,37,38]. SP-D has been implicated as a biomarker of chronic periodontitis due to its high concentration in plasma during the disease period [33]. The human SP-D gene is present on chromosome 10 (10q2.2-10q23.1) in the long arm with seven coding exons. About ninety-five SNPs have been reported in the SP-D gene with most located in the intronic region, while only eight SNPs have been found in the coding region, including rs7851984 (G/A), rs721917 (C/T), rs2839718 (G/C), rs7878336 (G/C), rs2243639 (A/G), rs3088308 (A/T), rs4469829 (A/G), and rs17851983 (A/G).

The genetic variations of the SP-D gene interrupt the normal regulation of homeostasis [39,40,41]. A report suggested a significant association of SP-D SNP (rs721917) and SP-D circulating levels with the allelic variant (Met11) is found associated with higher SP-D levels [42,43]. Contrarily, higher frequencies of both the alleles (Met11/Thr11) were detected in the population of North Europe [44]. However, several reports suggested the association of Thr11 allele with disease onset [41]. Many studies have reported the correlation of SP-D polymorphisms with the enhanced disease susceptibility or risk, such as lung fibrosis [45], cystic fibrosis [46], acute respiratory distress syndrome [47], neonatal respiratory distress syndrome [48,49], chronic obstructive pulmonary disease (COPD) [50], asthma [51], dysplastic and neoplastic pulmonary disorders [48,52], acute kidney injury [53], and gestational diabetes mellitus [54]. However, there is no study available that analyzes the association of SP-D polymorphisms with RAS onset. Therefore, this study was designed to investigate the association of SP-D SNPs with RAS development and also its risk factors.

## 2. Materials and Methods

### 2.1. Study Design and Setting

This study was carried out as a case-control study at Department of Biomedical Sciences, Advance Research Center for Biomedical Sciences (ARCBS), King Edward Medical University and de’Montmorency College of Dentistry, Lahore, Pakistan. The study was approved by the Institutional Review Board of the King Edward Medical University, Lahore, Pakistan via No. 83/RC/KEMU, dated 16 September 2016. Informed consents were obtained from all study participants.

### 2.2. Sample Collection

A total of 212 blood samples (106 RAS cases, 106 controls) were collected during 2019 from the routine diagnostic facility of Punjab Dental Hospital, Lahore, Pakistan. Data collection proforma was developed to establish RAS diagnosis as described previously [55]. Briefly, types of RAS (minor, major, and herpetiform) were recorded in the form of number, size, and site of oral mucosa. Study subjects with smoking histories were assessed by number of cigarette packs consumed per day. Body mass index (BMI) was calculated manually using Quetelet’s Index. Weight was measured (to the nearest to 0.5 kg after correcting zero error) using a standard spring balance and ensuring no shoes and the least amount of clothing were worn (without jackets and sweaters) by participants. Height was measured in the standing position by using a stadiometer without footwear, measuring to the nearest 0.1 cm and then units were converted to meters. BMI was taken as normal (18.5–24.9), underweight (<18.5), and obese (>25) [56]. Study subjects with COPD, asthma, acute infection, inflammatory bowel disease, and severe anemia and patients taking systemic steroids were excluded due to the potential role of SP-D in the onset of diseases. The data collection proforma is given in Table S1.

### 2.3. SP-D SNP Genotyping by Restriction Fragment Length Polymorphism (RFLP)

Total genomic DNA was isolated from whole blood using the GeneJET Whole blood genomic DNA purification kit (catalog # K0781, Thermo Fischer Scientific, Waltham, MA, USA). SNPs of SP-D gene (rs3088308, rs721917, rs2243639) were investigated by RFLP. The characteristics of SP-D SNPs are given in Table 1.

SNPs were amplified by polymerase chain reaction (PCR) in a thermal cycler (ProFlex PCR system, Thermo Fischer Scientific, Waltham, MA, USA). PCR reaction mixture of 50 μL consisted of 25 μL of 2 X PCR Master Mix (catalog # K0171, Thermo Fischer Scientific, Waltham, MA, USA), 10 pM of each primer, 2 ng of DNA, and dH2O up to 50 μL. The sequence of primers used for PCR amplification, amplicon size, and annealing temperature were as follows [57]: rs3088308 forward primer: 5′-ACG GAG GCA CAG CTG CTG-3′, reverse primer: 5′-GGA AAG CAG CCT CGT TCT-3′, 115 bp, 52 °C, rs721917 forward primer: 5′-CCC CAT AGC AGA GGA CAG AA -3′, 238 bp, 55 °C, reverse primer: 5′-CCA GGG TGC AAG CAC TGG AC-3′ and rs2243639 forward primer: 5′-CCC CAC TTC TCT CTC TGA CC-3′, reverse primer: 5′-CTG CTC ACC TGC TGC CCC CG-3′, 238 bp, 50 °C. Sterile nuclease-free water was used as the negative control. Agarose gel (1.5–2%) was used to resolve and analyze the PCR products. PCR products were purified using the GeneJET PCR purification kit (catalog # K0701, Thermo Fischer Scientific, Waltham, MA, USA). The purified PCR product was further subjected to RFLP analysis. Specified restriction enzymes and conditions used for SNP detection are given in Table 2. Restriction fragments were resolved using 12% polyacrylamide gel electrophoresis (PAGE) followed by staining with SYBR safe DNA gel stain (Thermo Fischer Scientific, Waltham, MA, USA). Gel was run at 80 V for 2 h and visualized in a gel documentation system (Bio-Rad, Hercules, CA, USA).

### 2.4. Statistical Analysis

All statistical analyses were conducted using the Statistical Package for Social Sciences software (SPSS 26). Categorical data were presented as frequency and percentage. The Hardy–Weinberg equilibrium was used for the control and RAS groups. Chi-square test was applied to calculate significance between genotype distribution and clinical parameters. Odds ratio (OR) and 95% confidence interval (CI) were used to estimate the strength of association of parameters and calculated by MedCalc OR calculator (version 20.218; https://www.medcalc.org/calc/odds_ratio.php, (accessed on 3 March 2021)). *p*-value ≤ 0.05 was considered to be statistically significant. The Adobe Illustrator CC 2018 program was used for labeling of PAGE gels.

## 3. Results

### 3.1. Patient Characteristics and RAS Establishment

Of the total subjects, females (118/212, 55.7%) constituted slightly higher than males (94/212, 44.3%). Within cases, the frequency of female subjects was higher (74/106, 69.8%) as compared to males (32/106, 30.1%). Demographic data showed that most of the study subjects were adults; >18 years of age with mean age of 29 years. The most abundant RAS population was students (38/106, 35.8%) and unemployed subjects (38/106, 35.8%). Minor aphthous (75.5%, *n* = 80) was the most common type of ulcer followed by herpetiform (21.7%, *n* = 23) and major aphthous (2.8%, *n* = 3) with subjects having five ulcers on average. Ulcer sizes ranged from 1 mm to 20 mm with 87.7% (*n* = 93) of them ≤5 mm size. Buccal mucosa (48.1%, *n* = 51) and labial mucosa (36.8%, *n* = 39) were the common sites involved in the RAS, while the less common sites included tongue (9.4%, *n* = 10), palate (3.8%, *n* = 4), and floor of mouth (1.9%, *n* = 2). More than three episodes of RAS in the past three years were reported in 90.5% (*n* = 96) of cases, occurring at different sites whose manipulation resulted in exacerbation of the ulcer. Topical or systemic steroids were used for ulcerations in 41.5% (*n* = 44) of cases. Irregular recurrence patterns occurred in the majority of patients (89.6%, *n* = 95), while 59.4% (*n* = 63) reported triggering factors, such as local trauma, infections, drugs, or specific food products. It was observed that 35.9% (*n* = 38) of cases were either non-smokers or developed RAS after quitting smoking, 5.6% (*n* = 6) were current smokers, 4.2% betel nut chewing, 3.8% tobacco snuffing, and 2.4% tobacco chewing. Ferritin, iron, zinc, folate, or vitamin B deficiency were observed in 25% cases, and low hemoglobin (Hb) levels were found in 34% (*n* = 36) cases. Total leukocyte counts ranged from 4.5 to 6.0 (25.5%, *n* = 27), 6.1 to 8.5 (44.3%, *n* = 47), and 8.6 to 10.5 (31.1%, *n* = 32) with a mean of 6.17. Body mass index (BMI) analysis showed that 40.6% of RAS subjects were obese, and 15.1% were underweight. The records of family history demonstrated that 70% (*n* = 75) of cases had a positive history of RAS in their families.

### 3.2. Association Analysis of SP-D SNPs

**Figure 1:**
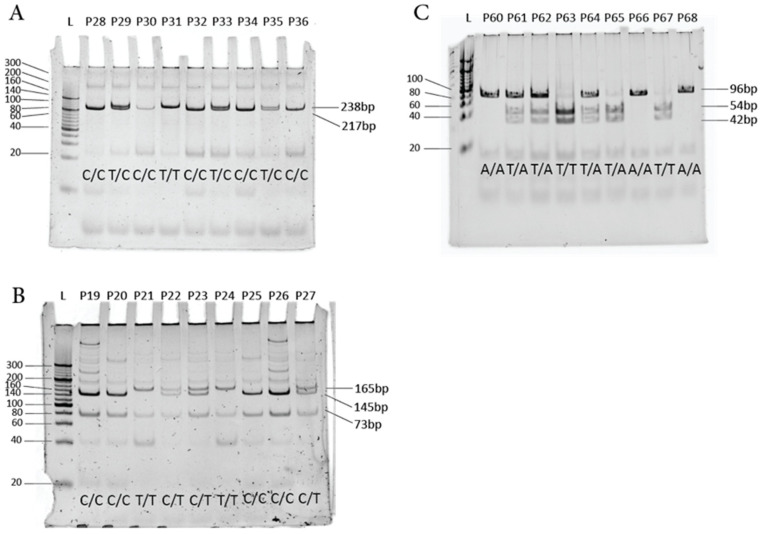
PAGE (12%) for detection of SP-D SNPs. L represents DNA ladder 50 bp, P represents patient number. (**A**) SP-D SNP rs721917, (**B**) SP-D SNP rs2243639, (**C**) SP-D SNP rs3088308.

SP-D SNPs (rs2243639, rs721917, and rs3088308) were detected by PCR-RFLP followed by PAGE. The PAGE results are given in Figure 1.

The genotype distribution of SNP rs2243639 analysis showed that the frequency of heterozygous variant C/T was higher in RAS patients (33.96% cases versus 23.58% controls). In the case of SNP rs721917, higher frequencies of T/C and T/T variants were present in the RAS group (T/C: 47.17% cases versus 35.85% controls, T/T: 9.43% cases versus 1.89% controls). SNP rs3088308 analysis showed that heterozygous variant T/A frequency was higher in the RAS group (49.06% cases versus 25.47% controls). Association analysis showed that SNP rs3088308 heterozygous T/A (95% (Cl): 1.57–5.03, *p*-value = 0.0005), homozygous variant A/A (95% (Cl): 1.8–6.7, *p*-value = 0.0002), T allele (95% (Cl): 1.09–2.36, *p*-value = 0.01), and A allele (95% (Cl): 1.42–3.91, *p*-value = 0.01), while SNP rs721917 homozygous variant T/T (95% (Cl): 1.15–25.35, *p*-value = 0.03) and T allele (95% (Cl): 1.28–3.10, *p*-value = 0.002) were significantly associated with the RAS. However, no association was observed for SNP rs2243639. The details of SP-D SNPs distribution among RAS and the control groups are given in Table 3. Furthermore, we observed that all genotype frequencies follow the Hardy–Weinberg equilibrium. The results are given in Table S2.

Genotype and allele type association analysis of SNP rs3088308 with gender demonstrated that genotypes T/A (OR = 5.06, 95% (CI): 1.89–15.7, *p*-value = 0.001), T/T (OR = 6.25, 95% (Cl): 1.52–11.9, *p*-value = 0.005), A allele (OR = 4.00, 95% (Cl): 1.65–7.58, *p*-value < 0.001), and T allele (OR = 5.7, 95% (Cl): 1.4–10.1, *p*-value < 0.001) were significantly associated with female gender in case subjects. While no association was found for Hb level, BMI, and RAS types. The results of case subjects are given in Table 4. On the other hand, the analysis of control subjects showed that T/A genotype (OR = 4.3, 95% (CI): 1.05–17.2, *p*-value = 0.04) was associated with low levels of Hb.

Association analysis of SNP rs721917 showed that genotype T/T (OR = 6.6, 95% (CI) = 1.3–33, *p*-value = 0.02) was associated with obese BMI range. While T/C (OR = 2.1, 95% (CI) = 0.9–4.6, *p*-value = 0.05) was found associated with normal BMI range in case subjects. While no association was found for gender, Hb level, and RAS types. The detailed results of case subjects are given in Table 5. However, T allele (OR = 2.3, 95% (CI) = 1.1–4.8, *p*-value = 0.02) was associated with male control subjects.

Furthermore, no association of SNP rs2243639 was found in case subjects. However, C/T allele (OR = 10, 95% (CI) = 2.6–44.9, *p*-value = 0.001) and T allele (OR = 4.5, 95% (CI) = 1.8–11.09, *p*-value = 0.001) were associated with the obese BMI range in control subjects, while C/C (OR = 21.6, 95% (CI) = 2.6–175, *p*-value = 0.003) and C allele (OR = 3.5, 95% (CI) = 1.5–8.2, *p*-value = 0.002) were associated with the normal BMI range in control subjects. The detailed results of case subjects are given in Table 6.

## 4. Discussion

RAS is a multifactorial chronic ulcerative disease with severely inflamed oral mucosa, and it is generally characterized by the presence of painful ulcerations of the oral mucosa. RAS pathogenesis remains unclear as multiple factors can cause its occurrence. Therefore, differential diagnosis of RAS is usually established for treatment regime [7]. Keeping in view that patients with positive family history are at higher risk to develop recurrent ulcerations [58,59] and not much data are available from Pakistan about the association of polymorphism with RAS, the current study was designed to investigate SP-D SNP association with RAS in the Pakistani population.

The results obtained are in agreement with the previous studies [60,61,62] that female subjects are at higher risk to develop oral ulcerations. In our study, most of the subjects were adults; older than 18 years of age with the mean age being 29 years. Another study from Pakistan reported mean age of 22 years for RAS onset [62]. Other studies reported the RAS onset at the peak age of 19 years and continued for their whole lives [63,64]. We observed that 35.9% of the RAS cases were either non-smokers or developed aphthous after quitting smoking, as previously described [65,66,67,68]. The buccal mucosa was the most common site involved for ulcer development. Similarly, buccal mucosa was reported to be the most common site for RAS development [64], while another study reported that the tongue was the predominant site followed by buccal mucosa and lower lip [69,70]. We noticed that 41.5% of participants had to use either a topical or systemic steroid for their aphthous ulcerations. However, it was shown that about 67.72% aphthous ulcers require no treatment, and stress was the major factor that contributed to RAS development [64,71]. Similarly, stress was considered a major factor in another study from Pakistan [26,62]. Normal Hb levels were detected in 78.8% of study participants. However, low Hb levels in the RAS group (42%) were reported from Pakistan [61,72], while normal Hb levels were suggested in another study [73]. During our research, hematinic deficiencies were seen in a small population of RAS cases that is lower as compared to other studies, such as ferritin deficiency [16,72,74,75], altered zinc levels [76,77,78,79,80,81], and B12 deficiency [82,83,84] in RAS patients. Moreover, a significant association was observed among Hb, iron, B12, and folic acid deficiencies and RAS occurrence [85]. Our study did not find any BMI differences in the RAS group; similarly, another study reported that all BMI groups have equal chances of RAS development [85,86,87,88,89]. However, in another study, the low BMI group has been found to be associated with RAS onset [90].

In the current investigation, the records of family history of RAS showed that 70% of cases had positive family history. Previously, 24% to 46% of RAS cases were reported with positive familial predisposition [91]. Genetic analysis of RAS showed that monozygotic twins had higher risks of developing the condition than dizygotic ones, thus indicating that genetic factors have the ability to modify the RAS susceptibility and severity, such as DNA polymorphism of interleukins (IL-2, IL-1β, IL-4, IL-5, IL-6, IL-8, IL-10) and TNFα [14,58,92]. In the Chinese population, a strong association of RAS with HLA-DRw9 has been documented [93].

SPs are considered critical for initiating the mucosal immune responses and play an important role in regulating the inflammatory responses [34]. It was reported that SP-D is expressed in the oral mucosa to protect it from a harmful environment [94,95]. Several studies suggested the presence of allelic variations of SP-D SNP rs721917 in different diseases, indicating that both variants may facilitate disease onset, such as allergic rhinitis (Thr11) [96], coronary stenosis (Thr11) [97], COPD (Thr11) [98], COPD (Met11) [39], type II diabetes (Met11) [99], lung cancer (Thr11) [100], rheumatoid arthritis (Thr11) [101], tuberculosis (Thr11) [102], atherosclerosis (Thr11) [53], cystic fibrosis (Thr11) [103], and multi-organ dysfunction syndrome (Thr11) [104]. SP-D SNP rs2243639 is known to be associated with the decreased risks of acute respiratory failure [105], Crohn’s disease [106], and ulcerative colitis [107] and COPD risk in the Caucasian population [108]. However, only few reports are available regarding the SNP rs3088308 association with disease susceptibility, such as COPD in smokers [109]. Currently, no data on associations of SP-D SNPs (rs721917, rs2243639, rs3088308) with RAS are available.

Our results showed that SNP rs3088308 heterozygous variant T/A, homozygous variant A/A, T allele, A allele and SNP rs721917 homozygous variant T/T and T allele were significantly associated with the RAS onset. However, no association was observed for SNP rs2243639. On the other hand, SNP rs3088308 genotypes T/A, T/T, A allele, and T allele were significantly associated with female gender, while SNP rs721917 genotype T/T was associated with the obese BMI range. In conclusion, we are reporting, for the first time, the association of SP-D rs3088308 and rs721917 with RAS.

## Figures and Tables

**Table 1:** Characteristics of SP-D SNPs used for genotyping of RAS.

SNP ID	rs2243639	rs721917	rs3088308
SNP type	Missense variant	Missense variant	Missense variant
Exon involved	Fourth exon	First exon	Seventh exon
Position	Chr10: 79941966 (GRCh38.p13)	Chr10: 79946568 (GRCh38.p13)	Chr10: 79938112 (GRCh38.p13)
Cytogenetic location	10q22.3	10q22.3	10q22.3
Natural Variant #	VAR_020939	VAR_020937	VAR_020940
Alleles	T > C/T > G forward strand	A > G forward strand	A > T forward strand
cDNA position	581	135	911
Protein position	180	11	290
Variation Type	Single nucleotide	Single nucleotide	Single nucleotide
Amino acid substitution	Thr180Ala	Met11Thr	Ser290Thr

**Table 2:** Restriction enzyme and RFLP conditions for SNP detection.

SNP	Restriction Enzyme	Genotype	Product Size (bp)	Incubation Temperature/Time
rs2243639	BstU1	T/T (wild type)	165, 73	60 °C/2 h
		C/C	145, 73	
		T/C	165, 145, 73	
rs721917	Tail	T/T (wild type)	238	37 °C/15 min
		C/C	217, 21	
		T/C	238, 217, 21	
rs3088308	MnII	T/T (wild type)	54, 42, 19	37 °C/15 min
		A/A	96, 19	
		T/A	96, 54, 42, 19	

**Table 3:** Distribution of SP-D SNPs genotypes and alleles among cases and controls.

SNP ID	Genotypes	Case *n* (%)	Control *n* (%)	OR	95% (CI)	*p*-Value
rs2243639	C/T	36 (33.9)	25 (23.6)	1.67	0.91–3.04	0.09
	T/T	9 (8.5)	15 (14.2)	0.56	0.23–1.34	0.19
	C/C	61 (57.5)	66 (62.3)	0.82	0.47–1.42	0.48
	C allele	158 (74.5)	157 (74.1)	1.02	0.66–1.5	0.91
	T allele	54 (25.5)	55 (25.9)	0.97	0.63–1.5	0.91
rs721917	T/C	50 (47.2)	38 (35.8)	1.5	0.92–2.77	0.09
	T/T	10 (9.4)	2 (1.8)	5.4	1.15–25.35	0.03
	C/C	46 (43.4)	66 (62.3)	0.39	0.22–0.69	0.003
	C allele	142 (66.9)	170 (80.2)	0.50	0.32–0.78	0.002
	T allele	70 (33.0)	42 (19.8)	1.99	1.28–3.10	0.002
rs3088308	T/A	52 (49.1)	27 (25.5)	2.8	1.57–5.03	0.0005
	T/T	35 (33.0)	35 (33.0)	1	0.56–1.77	1.0
	A/A	19 (17.9)	44 (41.5)	3.30	1.8–6.7	0.0002
	T allele	122 (57.5)	97 (45.7)	1.60	1.09–2.36	0.01
	A allele	90 (42.4)	115 (54.3)	1.62	1.42–3.91	0.01

**Table 4:** Association of SNP rs3088308 with study parameters in RAS subjects.

Study Parameters		T/A	T/T	A/A	A Allele	T Allele
		*n* (%)				
Gender	Female	36 (69.3) *p* = 0.001 Cl: 1.89–15.7	25 (71.4) *p* = 0.005 Cl: 1.52–11.9	12 (63.2) *p* = 0.1 Cl: 0.25–2.06	60 (66.7) *p* < 0.001 Cl: 1.65–7.58	86 (70.1) *p* < 0.001 Cl: 1.4–10.1
	Male	16 (30.7) *p* = 0.001 Cl: 0.42–0.92	10 (28.8) *p* = 0.005 Cl: 0.34–0.87	7 (36.8) *p* = 0.1 Cl: 0.48–3.86	30 (33.3) *p* < 0.001 Cl: 0.66 –0.96	36 (29.5) *p* < 0.001 Cl: 0.26–0.93
Hb level	Low	18 (34.6) *p* = 0.8 Cl: 0.47–2.36	11 (31.4) *p* = 0.3 Cl: 0.35–2.0	7 (36.8) *p* = 0.7 Cl: 0.41–3.27	32 (35.5) *p* = 0.6 Cl: 0.63–2.00	40 (32.8) *p* = 0.6 Cl: 0.49–1.56
	Normal	34 (65.4) *p* = 0.8 Cl: 0.42–2.11	24 (68.6) *p* = 0.6 Cl: 0.49–2.8	12 (63.1) *p* = 0.7 Cl: 0.30–2.40	58 (64.4) *p* = 0.6 Cl: 0.49–1.56	82 (67.2) *p* = 0.6 Cl: 0.63–2.00
BMI	Underweight	8 (15.4) *p* = 0.3 Cl: 0.46–5.04	3 (8.6) *p* = 0.5 Cl: 0.18–2.88	2 (10.5) *p* = 0.7 Cl: 0.14–3.64	12 (13.3) *p* = 0.6 Cl: 0.52–2.70	14 (11.4) *p* = 0.6 Cl: 0.36–1.92
	Normal	24 (46.2) *p* = 0.9 Cl: 0.34–1.66	15 (42.8) *p* = 0.6 Cl: 0.52–3.01	10 (52.6) *p* = 0.5 Cl: 0.42–3.18	44 (48.9) *p* = 1.0 Cl: 0.69–2.08	54 (44.3) *p* = 0.7 Cl: 0.48–1.43
	Obese	20 (38.5) *p* = 0.8 Cl: 0.48–2.49	10 (28.6) *p* = 0.3 Cl: 0.36–2.24	7 (36.8) *p* = 0.8 Cl: 0.34–2.74	34 (37.8) *p* = 0.3 Cl: 0.43–1.33	54 (44.3) *p* = 0.7 Cl: 0.75–2.28
RAS types	Minor	42 (80.7) *p* = 0.2 Cl: 0.71–4.36	25 (71.4) *p* = 0.9 Cl: 0.28–1.82	13 (68.4) *p* = 0.4 Cl: 0.21–1.92	68 (75.5) *p* = 0.9 Cl: 0.53–1.89	92 (75.4) *p* = 0.9 Cl: 0.52–1.86
	Major	-	2 (5.7) *p* = 0.1 Cl: 0.49–228.5	-	-	4 (3.3) *p* = 0.1 Cl: 0.336–129.3
	Herpetiform	10 (19.3) *p* = 0.4 Cl: 0.27–1.70	8 (22.8) *p* = 0.8 Cl: 0.38–2.67	6 (31.6) *p* = 0.3 Cl: 0.59–5.30	22 (24.5) *p* = 0.5 Cl: 0.62–2.28	26 (31.3) *p* = 0.5 Cl: 0.43–1.59

**Table 5:** Association of SNP rs721917 with study parameters in RAS subjects.

Study Parameters		T/C	T/T	C/C	C Allele	T Allele
		*n* (%)				
Gender	Female	32 (64.0) *p* = 0.3 Cl: 0.28–1.48	8 (80.0) *p* = 0.4 Cl: 0.38–9.5	33 (71.8) *p* = 0.5 Cl: 0.54–2.93	98 (69.0) *p* = 0.9 Cl: 0.55–1.89	48 (68.6) *p* = 0.9 Cl: 0.52–1.81
	Male	18 (36.0) *p* = 0.3 Cl: 0.007–0.03	2 (20.0) *p* = 0.4 Cl: 0.10–2.61	13 (28.2) *p* = 0.6 Cl: 0.34–1.81	44 (31.0) *p* = 0.9 Cl: 0.52–1.81	22 (31.4) *p* = 0.9 Cl: 0.55–1.89
Hb level	Low	15 (30.0) *p* = 0.4 Cl: 0.31–1.60	5 (50.0) *p* = 0.2 Cl: 0.56–7.78	16 (34.8) *p* = 0.8 Cl: 0.47–2.39	47 (33.0) *p* = 0.7 Cl: 0.48–1.62	25 (35.7) *p* = 0.7 Cl: 0.61–2.04
	Normal	35 (70.0) *p* = 0.4 Cl: 0.62–3.12	5 (50.0) *p* = 0.2 Cl: 0.12–1.77	30 (65.2) *p* = 0.8 Cl: 0.41–2.10	95 (67.0) *p* = 0.7 Cl: 0.61–2.04	45 (64.3) *p* = 0.7 Cl: 0.48–1.62
BMI	Underweight	6 (12.0) *p* = 0.07 Cl: 0.29–3.05	-	7 (15.2) *p* = 0.4 Cl: 0.50–5.18	20 (14.0) *p* = 0.1 Cl: 0.72–4.98	6 (8.6) *p* = 0.1 Cl: 0.20–1.37
	Normal	28 (56.0) *p* = 0.05 Cl: 0.9–4.6	2 (20.0) *p* = 0.1 Cl: 0.05–1.29	19 (41.3) *p* = 0.2 Cl: 0.32–1.52	66 (46.5) *p* = 0.5 Cl: 0.66–2.12	32 (45.7) *p* = 0.5 Cl: 0.47–1.50
	Obese	16 (32.0) *p* = 0.06 Cl: 0.21–1.03	8 (80.0) *p* = 0.02 Cl: 1.3–33	20 (43.5) *p* = 0.7 Cl: 0.52–2.51	46 (32.5) *p* = 0.1 Cl: 0.35–1.14	32 (45.7) *p* = 0.1 Cl: 0.87–2.84
RAS types	Minor	36 (72.0) *p* = 0.4 Cl: 0.28–1.70	7 (70.0) *p* = 0.6 Cl: 0.17–3.07	37 (80.4) *p* = 0.5 Cl: 0.64–4.07	110 (77.5) *p* = 0.3 Cl: 0.71–2.63	50 (71.4) *p* = 0.3 Cl: 0.37–1.39
	Major	1(2.0) *p* = 0.9 Cl: 0.06–18.43	1 (10.0) *p* = 0.1 Cl: 0.60–3.37	-	1 (0.7) *p* = 0.1 Cl: 0.02–1.55	3 (4.3) *p* = 0.1 Cl: 0.64–1.82
	Herpetiform	13 (26.0) *p* = 0.4 Cl: 0.57–3.58	2 (20.0) *p* = 0.4 Cl: 0.16–4.25	9 (19.6) *p* = 0.8 Cl: 0.28–1.85	31 (31.8) *p* = 0.6 Cl: 0.44–1.71	17 (24.3) *p* = 0.6 Cl: 0.58–2.25

**Table 6:** Association of SNP rs2243639 with study parameters in RAS subjects.

Study Parameters		C/T	T/T	C/C	C Allele	T Allele
		*n* (%)				
Gender	Female	25 (69.4) *p* = 0.9 Cl: 0.43–2.48	9 (100) *p* = 0.2 Cl: 0.55–174	39 (64.0) *p* = 0.2 Cl: 0.2–1.3	123 (77.8) *p* = 0.7 Cl: 0.41–1.92	43 (79.6) *p* = 0.7 Cl: 0.51–2.38
	Male	11 (30.6) *p* = 0.9 Cl: 0.40–2.29	-	22 (34.0) *p* = 0.2 Cl: 0.24–1.35	35 (22.2) *p* = 0.7 Cl: 0.51–2.38	11 (20.4) *p* = 0.7 Cl: 0.41–1.92
Hb level	Low	12 (33.3) *p* = 0.9 Cl: 0.40–2.24	20 (32.8) *p* = 0.7 Cl: 0.39–1.99	4 (44.4) *p* = 0.4 Cl: 0.40–6.46	52 (33.0) *p* = 0.5 Cl: 0.43–1.58	20 (37.0) *p* = 0.5 Cl: 0.62–2.28
	Normal	24 (66.7) *p* = 0.9 Cl: 0.44–2.44	41 (67.2) *p* = 0.7 Cl: 0.50–2.54	5 (55.6) *p* = 0.4 Cl: 0.15–2.44	106 (67.0) *p* = 0.5 Cl: 0.62–2.28	34 (63.0) *p* = 0.5 Cl: 0.43–1.58
BMI	Underweight	3 (8.3) *p* = 0.4 Cl: = 0.15–2.43	8 (13.1) *p* = 0.9 Cl: 0.43–5.49	1 (11.1) *p* = 0.9 Cl: 0.11–8.57	19 (12.0) *p* = 0.5 Cl: 0.47–3.78	5 (9.2) *p* = 0.5 Cl: 0.26–2.10
	Normal	17 (47.2) *p* = 0.8 Cl: 0.42–2.11	30 (49.2) *p* = 0.7 Cl: 0.51–2.39	4 (44.4) *p* = 0.9 Cl: 0.27–4.30	77 (48.7) *p* = 0.7 Cl: 0.59–2.04	25 (46.3) *p* = 0.7 Cl: 0.48–1.68
	Obese	16 (44.5) *p* = 0.5 Cl: 0.56–2.87	23 (37.7) *p* = 0.4 Cl: 0.34–1.65	4 (44.4) *p* = 0.8 Cl: 0.24–3.91	62 (39.2) *p* = 0.5 Cl: 0.43–1.50	24 (44.5) *p* = 0.5 Cl: 0.66–2.31
RAS types	Minor	25 (69.4) *p* = 0.3 Cl: 0.24–1.54	6 (66.7) *p* = 0.5 Cl: 0.15–2.79	49 (80.4) *p* = 0.1 Cl: 0.75–4.5	123 (77.8) *p* = 0.1 Cl: 0.81–3.20	37 (68.5) *p* = 0.1 Cl: 0.311–1.23
	Major	1 (2.8) *p* = 0.6 Cl: 0.11–32.4	-	1 (1.6) *p* = 0.8 Cl: 0.04–12.04	3 (1.9) *p* = 0.9 Cl: 0.10–10.1	1 (1.8) *p* = 0.9 Cl: 0.09–9.5
	Herpetiform	10 (27.8) *p* = 0.3 Cl: 0.60–3.92	3 (33.3) *p* = 0.4 Cl: 0.41–7.8	11 (18.0) *p* = 0.1 Cl: 0.35–1.20	32 (20.3) *p* = 0.1 Cl: 0.30–1.22	16 (29.6) *p* = 0.1 Cl: 0.81–3.31

## Data Availability

The data used to support the findings of this study are available from the corresponding authors upon request.

## Supplementary Materials

The following supporting information can be downloaded at: https://www.mdpi.com/article/10.3390/genes14051119/s1, Table S1: Data collection proforma used during sampling; Table S2: SP-D SNPs genotype distribution in study population by HWE.
