# Supplementary material for: Association of Surfactant Protein D Single Nucleotide Polymorphisms rs721917, rs2243639, rs3088308 with Recurrent Aphthous Stomatitis in Pakistani Population
Source: Genes (Basel). 2023 May 22;14(5):1119. doi: 10.3390/genes14051119 (PMC10218356; doi:10.3390/genes14051119)
Supplement: Supplementary file 1 [file genes-14-01119-s001.zip › genes-2393548-supplementary.pdf]

Table S1: Data collection proforma used during sampling

Patient Number --------------------------- Date of Enrollment ---------------------------			
A- Demographic data			
A-1	File Number		
A-2	Name of patient		
A-3	Father's name		
A-4	NIC number		
A-5	Current Address		
A-6	Contact number		
A-7	Occupation		
A-8	Age		
A-9	Gender	Male	Female
B- Inclusion Criteria			
Fulfills all 4 major criteria		Yes	No
B-1	Single or multiple round/oval ulcers, shallow, regular margins, yellow-gray base, surrounded by erythematous margins, never preceded by vesicles, less than 1 cm in diameter	Yes	No
B-2	At least 3 attacks of RAS within past 3 years, ulcers do not appear in the same focal site	Yes	No
B-3	Painful lesion exacerbated by movement of ulcer affected area	Yes	No
B-4	Ulcer heals spontaneously without squeal, with or without treatment	Yes	No
Fulfills at least 1 minor criteria		Yes	No
B-5	Family history of RAS	Yes	No
B-6	First attack below 40 years of age	Yes	No
B-7	Non keratinized oral mucosa involved	Yes	No
B-8	Ulcer lasts few days to few weeks	Yes	No
B-9	Irregular recurrence pattern	Yes	No
B-10	Non-specific inflammation on histopathology.	Yes	No
B-11	Triggered by hormonal changes, specific foods, drugs, infections or local trauma	Yes	No
B-12	Documented deficiency of Ferritin, Iron, Folate, B-12, Zinc	Yes	No
B-13	Non smoker or RAS developed after quitting smoking	Yes	No
B-14	Heals with oral or systemic steroids	Yes	No
C- Exclusion Criteria			
C-1	History of COPD	Yes	No
C-2	History of Asthma	Yes	No
C-3	Severe anemia (< 8 gm/dl)	Yes	No
C-4	Inflammatory Bowel Disease	Yes	No
C-5	Acute Infections	Yes	No
C-6	Systemic Steroids	Yes	No

D- Ulcer Examination				
D-1	Type	Minor	Major	Herpetiform
D-2	Number of ulcers			
D-3	Size (mm)			
D-4	Site			
E- Additional Info				
E-1	Height (m)			

E-2	Weight (kg)			
E-3	Body mass index (BMI kg/m^2)			
E-4	Smoking Status	Never smoker	Current smoker	Ex-smoker
E-5	Pack years of smoking			
E-6	Pan chewing	Yes		No
E-7	Niswar chewing	Yes		No
E-8	Gutka chewing	Yes		No
F- Tests				
F-1	Hemoglobin			
F-2	Total leukocyte count			

Table S2: SP-D SNPs Genotypes distribution in study population by HWE

SNP ID	Recessive allele (q)	Frequency of recessive allele (q^2)	Frequency of dominant allele (1-q=p)	HWE $1=p^2+2pq+q^2$
rs3088308				
Case	0.179	0.032	0.821	1.0
Control	0.415	0.172	0.585	1.0
rs721917				
Case	0.434	0.188	0.566	1.0
Control	0.627	0.393	0.373	1.0
rs2243639				
Case	0.575	0.330	0.425	1.0
Control	0.623	0.388	0.377	1.0
